# Lectures on the Diagnosis and Treatment of Diseases of the Lungs

**Published:** 1841-01-30

**Authors:** W. W. Gerhard


### MEDICAL EXAMINER.

DEVOTED TO MEDICINE, SURGERY, AND THE COLLATERAL SCIENCES.

No. 5.] PHILADELPHIA, SATURDAY, JANUARY 30, 1841. [Vol. IV.

### LECTURES ON THE DIAGNOSIS AND TREATMENT OF DISEASES OF THE LUNGS.

BY W. W. GERHARD, M. D.

### LECTURE XIII.—(*Continued.*)

PULMONARY PHTHISIS—MODE OF ATTACK.

Pulmonary phthisis, like other forms of tuberculous diseases, occurs either as an acute or chronic affection. A certain number of symptoms are common to both varieties; but others are peculiar to each, or at least are so much modified that it is difficult at times to recognise the identity of the two affections. The acute disease is attended by much febrile excitement, and by the general characters of an inflammatory affection. Indeed, it is either connected with an ordinary inflammation, or the secretion of the tuberculous substance itself is but little different from that process by which the common products of inflammation are formed. In the chronic disorder the alteration is not of an inflammatory, or even of an active, secretory kind,—it is a slow change in the condition of the capillary vessels of the body.

Both the acute and chronic varieties may be attended with a local inflammatory action in the lungs, or may be almost entirely free from it. In the latter case the lungs are merely involved as a part of the general disorder which shows itself in these organs, from their structure being favourable to the tuberculous deposit. When the disease is complicated with local inflammation, this may precede, accompany, or follow the tuberculous secretion. In the acute variety the inflammation generally attacks the serous membranes, and in the chronic the mucous, although this is not always the case, for the inflammation of any tissue of the lung may be closely connected with the abnormal formation. There has been much confusion of ideas on this subject from the great variety in the connection which often exists between inflammation and tubercle; this is very similar to the connection between the local disease and the general diathesis; in fact, the complicating local disease is almost invariably of an inflammatory character, so that the question is at last almost narrowed down to this—is inflammation the cause of tubercles in the lungs, and we may say in the other organs, though this is not immediately connected with our subject? If you seek a reply to the naked question, you will be compelled to answer negatively,—but if you modify it so as to apply it to those varying conditions which are continually occurring in the human body, you will answer that it is one of the causes. That is, it will develope the disease very frequently in persons who present a strong tuberculous diathesis, and occasionally in those who do not. In the latter case especially, and to a certain extent in the former, it acts in two ways,—as a direct disturber of the lungs, and as a depressing agent upon the whole system. When inflammation occurs in this way before tubercles are positively developed, it may act during its continuance, and the tuberculous affection then coincides with the inflammatory action, or this may occur after the latter has terminated. It then acts chiefly as a disordering agent upon the general system, with a slight local determination to the part. In the former case, the local action of the cause is the predominant one.

The inflammation of different tissues does not, as I have stated, exert an equal agency upon the development of tubercle. To understand this, we must analyze them separately.

1. First of the serous membranes. Pleurisy is perhaps the most active of all these inflammations. Like the others, it attacks individuals in good health, or labouring simply under a scrofulous diathesis, and tubercles are developed during its course, or soon afterwards, or it coincides with the rapid formation of tubercles, which are then usually formed at the same time in the pleura, the false membranes, and the lungs proper, or it may occur as a mere secondary inflammation after the tubercles are formed, or are even tolerably advanced; in the latter case the pleurisy is a healthy, or at least a preservative inflammation, designed to prevent perforation of the pleura. All these varieties may be properly classed under the head of tuberculous pleurisy.

The first variety is the most difficult to distinguish, because the disease does not at first differ from ordinary pleurisy, and the important complication may be overlooked. The signs of the pleurisy are either gradually mingled with those of the tuberculous disease, or at least disappear when the symptoms of phthisis show themselves. In this case the pleuritic effusion is often extremely large, and the disease is then sometimes ascribed to the absorption of the empyema. The pus has undoubtedly an influence upon the formation of tubercles, but in most cases it acts merely as other causes of the disease,—that is, by producing an irritating action upon the part, and a general depressing influence on the whole body. The second variety is that in which tubercles are formed at the same time, and apparently by the same morbid action as the ordinary products of inflammation. The pleurisy is readily recognised; but the tuberculous complication can only be distinguished by careful attention to its symptoms, and even then the diagnosis is but a probable one. In the last variety there is, of course, no difficulty in ascertaining the nature of the pleurisy.

2. Bronchitis and pneumonia occasionally occur amongst the earliest lesions in the acute forms of phthisis. The bronchitis is then of the common mucous kind, and very rarely passes into tuberculous phthisis, except in those cases in which it is connected with a strong developed scrofulous diathesis. But the bronchial inflammation is extremely frequent as an early complication, coinciding with the first formation of tubercles, or following them. In the latter case it is most marked in the tubes which run through the clusters of tubercles, and it is then nothing but the ordinary secondary bronchitis, which gradually increases as the disease advances, and is most intense when softening has taken place, and the mucous membrane is irritated by the continual passage of the softened tubercles. Pneumonia is the least frequent of those local inflammations which act as determining causes of acute tubercles; it is rarely of the frank sthenic kind, but generally occurs in scattered lobules, bearing a close analogy to the lobular pneumonia of young children, or the variety of inflammation which attends the formation of metastatic abscess; it is, of course, difficult in these cases to decide, if the pneumonia is really antecedent to the tubercles, or occurs under the relation of a mere attendant, or even a secondary result.

In chronic cases of phthisis the preceding inflammation is usually of the bronchial variety; a common chronic mucous catarrh passing by insensible shades into pulmonary phthisis,—that is, a time arrives when the secretion of tuberculous matter takes place, and the bronchitis is no longer simple. This is not, however, the only inflammation which proves a determining cause of the more chronic forms of phthisis; pleurisy not unfrequently produces a like result, especially in those cases where the effusion has been large. Pneumonia rarely produces the same result; indeed, this inflammation is, on the whole, remarkably independent of tubercle.

*Phthisis without local inflammation at the commencement.* There is no doubt that many cases of phthisis, probably the larger number, originate without being preceded, or even at first accompanied by local inflammation; when this occurs, it is strictly secondary. These cases of the disease are sufficiently described in the commencement of this lecture, and in fact do not differ from those of general or constitutional tuberculous disease, except in the predominance of the disorder of the lungs. They may therefore be latent for a considerable time, and only attract attention to the lungs when the disease is sufficiently advanced to produce some secondary inflammation. The principal symptoms of the disease are therefore those of the general disorder, with or without the addition of the signs caused by the local mischief; these are not always developed sufficiently to attract much notice until the disease is quite decided. The mechanism of the pulmonary disorder, if such an expression can be used, is merely a direct secretion from the vessels, and is sometimes connected with a diminished, instead of an increased vascular action in the part, although this is not invariably the case even at first, and is very seldom so after the disease is developed.

*Symptoms.*—Phthisis is, or soon becomes so complicated a disorder, that a constant analysis is necessary in the study of the symptoms. If these are regarded in a crude, general way, they are often extremely indistinct; hence, many writers upon the subject content themselves with the signs of the disease as fully established when diagnosis is no longer a matter of doubt, or they add to this the general characters which are usually described as designating the scrofulous temperament. But as the discoveries of Laennec prepared the way both for a full understanding of the pathological characters of the advanced disease of the physical signs which attend them during life, physicians have not rested satisfied with this view of the subject, but have ascended, as it were, to the source of the affection, and have laboured to point out the initial steps, or at least the symptoms which occur very early in the disorder. Still you will find in many works on the subject, even of the most recent date, that the physical signs which occur sometimes quite late in the disease, are brought forward as indicative of the earliest stage of the disorder, which in most cases they certainly are not. We are obliged, therefore, to divide the symptoms into several different sorts, which will lead us naturally to the study of the connected or dependent diseases. 1. First we have a series of symptoms dependent upon the tubercular disease, considered as a general disorder. 2. Symptoms connected necessarily with the development of tubercles in the lungs, including, of course, the physical signs of the disorder, properly so called. 3. Symptoms dependent upon the accessary disease of the lungs and air-passages, including larynx and trachea, which are present to a greater or less degree, in nearly every case of the disease. 4. Symptoms of disorder of the organs, some of which depend upon a deposit of tubercle in the tissue, but for the most part are connected either with a positive inflammation or a mere functional disorder; to a greater or less extent these take place in most instances of phthisis. You do not, of course, expect to meet these symptoms in every case of the disease; many of them may be obscure, and some absent entirely; but we do in reality scarcely ever meet with a case in which they are all badly defined; that is, with a case of true latent phthisis; cases in which the disease is nearly latent, are quite common.

1. *General symptoms common to phthisis and other tuberculous diseases.* These differ in the acute and chronic varieties, in degree and to a certain extent, in nature. In the acute variety a rapid deposit of tuberculous matter takes place, generally throughout a number of organs at the same time; this approaches very nearly to an inflammatory secretion, and it is attended with a general disturbance of the body, which differs little from inflammatory fever, especially the fever which attends a sub-acute inflammation of the pleura or other serous membranes. The pulse is extremely frequent, generally from one hundred to one hundred and thirty in the minute, quick and jerking; these characters are often difficult to define, but are at the same time very well marked. The febrile excitement is continued, and does not cease during the twenty-four hours, diminishing a little in the morning, and becoming more intense towards the middle of the day; at night there is almost always sweating, which at times is extremely profuse, and as a general rule, is abundant. There are rarely chills, generally a mere sensation of chilliness at irregular times, and differing therefore from the chills of well-defined hectic, which occur in the latter stages of phthisis. The accessary symptoms, or those connected with the alimentary canal, are strictly such as would be supposed to exist in cases of high fever, such as thirst, anorexia, and constipation; but they are less severe than in most instances of febrile excitement, because the stomach and bowels do not at all participate in the earlier disturbance of the system. The general appearance and countenance of the patient change when the fever is developed. The expression is restless,—the lips and countenance pale and flushed at irregular times,—the flush is often circumscribed when the fever is most considerable, but the tint is of a much lighter red than in pneumonia. The flush is not peculiar to any one form of tuberculous fever, but occurs without reference to the part affected. The countenance is often indicative of much dyspnœa, with dilatation of the nostrils, if there be a very large and rapid secretion of tubercles. The emaciation is rapid, partly from a direct effect of the tuberculous disorder, and partly from the profuse sweats which rapidly enfeeble the patient.

These signs, in themselves, are not positively pathognomonic of acute tuberculous disease, but they scarcely occur in a high degree from any other cause,—not that all cases of acute tuberculization are necessarily attended with them in their highest degree, but you will find that they exist to a greater or less extent in nearly every case of the disorder, and that their value is much increased by the very slight development or entire absence of other lesions sufficient to account for the fever, especially if conjoined with one other character,—that is, persistence,—for this fever does not rapidly decline; on the contrary, it usually lasts for a considerable period, and then resists all treatment. Pleurisy of a sub-acute character approaches very nearly in the febrile symptoms to acute phthisis, whether the pleurisy be complicated or not with tubercles; in fact, I have little doubt that the pathological condition of the economy which attends the formation of the lymph, and that of acute tuberculous disease, differ but very slightly from one another. This, however, is not sustained by a course of demonstrative reasoning, and therefore is of little interest until it is better developed. It is the reasoning by exclusion which gives to the acute tuberculous fever a great portion of its value.

In the more chronic cases of phthisis the general signs of the disorder are more difficult to distinguish, because their development is slow, and the fever in the early stages is comparatively unimportant. The signs which are most decided are those indicative of a deterioration of the constitution and of the nutrition. The skin of the patient is generally of a dull tint; or if his complexion be naturally very clear, and the capillary circulation extremely active, the cheeks are from time to time flushed with a circumscribed redness, not very unlike that of acute phthisis, but less decided. At the same period, those peculiarities which are supposed to indicate a scrofulous or tuberculous constitution are often more developed,—that is, the blueness of the conjunctiva, and the rounded fusiform appearance of the ends of the fingers, which, although not peculiar to this condition of things, is certainly more common then than under any other circumstances. The moderately chronic cases are also accompanied with fever, which is often slight, and sometimes limited to a mere sensation of heat or burning at the palms of the hands and feet: the sensibility to cold is at the same time often much increased; but there is very rarely a distinct chill, except from inflammatory complication.

In short, the ordinary cases of phthisis offer as symptoms, emaciation and slight fever, with an increase of the peculiarities designating either a constitutional diathesis or a tendency to the disease from an original feebleness of constitution. The very chronic cases are more and more obscure as regards the general symptoms in proportion as the disease is slower in development. The addition of local signs of irritation confirms the value of the more chronic constitutional symptoms, as well as of the acute, provided these local symptoms do not disappear very readily.

